# Characteristics of cardiopulmonary exercise capacity in adults with different degrees of obesity

**DOI:** 10.3389/fphys.2024.1466153

**Published:** 2025-01-20

**Authors:** Shukun Deng, Shengrui Mei, Qunyan Zhou, Wenjun Zhi, Wenjun Wu, Junyan Cai, Peng Yuan

**Affiliations:** ^1^ Department of Rehabilitation Medicine, The Affiliated Wuxi People’s Hospital of Nanjing Medical University, Wuxi People’s Hospital, Wuxi Medical Center, Nanjing Medical University, Wuxi, China; ^2^ Department of Nutrition, The Affiliated Wuxi People’s Hospital of Nanjing Medical University, Wuxi People’s Hospital, Wuxi Medical Center, Nanjing Medical University, Wuxi, China; ^3^ Department of Endocrine, Jinshan Branch of Shanghai Sixth People’s Hospital, Shanghai, China; ^4^ Department of Rehabilitation Medicine, The Affiliated Hospital of Jiangnan University, Wuxi, China

**Keywords:** Obesity, cardiopulmonary exercise test, cardiopulmonary exercise capacity, breathing reserve, heart rate response, oxygen pulse

## Abstract

**Objective:**

To explore the characteristics of cardiopulmonary exercise capacity in adults with different degrees of obesity through cardiopulmonary exercise test (CPET).

**Methods:**

From September 2019 to January 2024, the data of patients undergoing CPET in the Rehabilitation Department of the Affiliated Wuxi People’s Hospital of Nanjing Medical University were analyzed retrospectively. A total of 231 cases were included. They were categorized into five groups based on their body mass index (BMI): the control group (18.5 ≤ BMI < 24 kg/m^2^, n = 28), the overweight group (24.0 ≤ BMI < 28 kg/m^2^, n = 48), the mild obesity group (28 ≤ BMI < 35 kg/m^2^, n = 75), the moderate obesity group (35.0 ≤ BMI < 40 kg/m^2^, n = 47), and the severe obesity group (BMI ≥ 40 kg/m^2^, n = 33). Collected informations on the age, gender, height, and weight of five groups of participants. The VO_2_ at anaerobic threshold (VO_2AT_), percentage of predicted VO_2AT_ (VO_2AT_% Pred), peak oxygen consumption (VO_2peak_), percentage of predicted VO_2peak_ (VO_2peak_% Pred), peak kilogram oxygen consumption (VO_2peak_/kg), maximum exercise power (WR_max_), breathing reserve (BR), maximum heart rate (HR_max_), percentage of predicted HR_max_ (HR_max_% Pred), maximum O_2_ pulse (VO_2_/HR_max_), percentage of predicted maximum O_2_ pulse (VO_2_/HR_max_%Pred), maximum relative O_2_ pulse (VO_2_/HR_max_/kg),heart rate response (HRr), forced vital capacity (FVC), ratio of forced expiratory volume to vital capacity in 1 s (FEV1/FVC), percentage of predicted forced vital capacity (FVC% Pred), percentage of predicted forced expiratory volume ratio of 1 s (FEV1% Pred), peak expiratory flow rate (PEF), maximum exercise ventilation (VE_max_), maximum voluntary ventilation (MVV) and other indicators during the CPET were collected. Single factor analysis of variance was used to compare the mean of each index between groups. Spearman correlation analysis was used to analyze the correlation between BMI and various indicators.

**Results:**

There was no statistical significance in gender composition, age, height, and exercise habit of the five groups of participants (P > 0.05). The body mass and BMI of the five groups had significant differences (P < 0.001). In terms of cardiopulmonary exercise capacity, there were statistical differences among the five groups in the overall distribution of VO_2AT_ (H = 37.370,P < 0.001), VO_2AT_/kg (H = 34.747, *P* < 0.001), VO_2peak_ (H = 23.018,P< 0.001), VO_2peak_/kg (H = 66.606, *P* < 0.001) and WR_max_%Pred (H = 45.136, *P* < 0.001). There was no significant difference among the five groups in the overall distribution of VO_2AT_%Pred, VO_2peak_%Pred and WR_max_. There were statistical significant difference among the five groups in HR_max_ (F = 2.443, *P* = 0.048), HR_max_%Pred (F = 6.920, *P* < 0.001), VO_2_/HR_max_ (F = 8.803, *P* < 0.001), VO_2_/HR_max_%Pred (F = 11.354, *P* < 0.001), VO2/HRmax/kg (F = 18.688, *P* < 0.001) and BR (F = 6.147, *P* < 0.001) and HRr (F = 9.467, *P* < 0.001). There were no significant differences among the five groups in RER_max_ (F = 0.336, *P* > 0.05). In terms of static pulmonary function, there were significant differences among the five groups in FVC%Pred (F = 4.577, *P* = 0.001), FEV1%Pred (F = 3.681, *P* = 0.006) and FEV1/FVC (F = 3.344, *P* = 0.011). There was no differences among the five groups in MVV(P> 0.05), and there were significant differences among the five groups in VE_max_ (*P* = 0.005) In terms of correlation analysis, BMI was positively correlated with VO_2AT_,VO_2peak,_ VE_max_ and VO_2_/HR_max_, and negatively correlated with VO_2AT_/kg, VO_2peak_/kg,WR_max_%Pred, HR_max_%Pred, VO_2_/HR_max_%Pred, VO_2_/HR_max_/kg,BR and HRr. In terms of static pulmonary function, BMI was negatively correlated with FVC%Pred, FEV1%Pred.

**Conclusion:**

With the aggravation of obesity, the maximum exercise ability of adults decreases, VO_2peak_/kg and VO_2_/HR_max_%Pred decreases, and the breathing reserve decreases.

## 1 Introduction

Obesity is a global public health problem (Gillison et al., 2022; Biener, Cawley, and Meyerhoefer, 2020; Jebeile et al., 2022). According to Chinese standards, approximately half of the adult population is overweight or obese, making China the nation with the highest number of individuals affected by overweight or obesity globally (Wang et al., 2021). In 2016, the American Society of Endocrinologists defined obesity as a disease (Garvey et al., 2016). According to the WHO, more than 4 million people die each year because of being overweight or obese. So, how is the cardiopulmonary exercise capacity of obese adults? Does the cardiopulmonary exercise ability of obese adults affect their mortality rate? Obesity can significantly increase the risk of type 2diabetes, hypertension, dyslipidemia, cardiovascular diseases and other diseases (Fingeret, Marques-Vidal, and Vollenweider, 2018; Kim, Lim, and Kim, 2021; Piché, Tchernof, and Després, 2020). Obesity is associated with an increased risk of various cancers, a point emphasized in the 2022 practice statement from the Obesity Medicine Association (OMA). According to a cohort study by Santamaría-Ulloa, each one-unit increment in BMI was significantly associated to a 3.1% and 2.6% increment in general and cardiovascular mortality respectively (Santamaría-Ulloa, Chinnock, and Montero-López, 2022). At present, the research on obesity mainly focuses on the exploration of therapeutic methods and the comparison of therapeutic effects (Chen et al., 2023). There is a dearth of research on the characteristics of cardiopulmonary function and exercise capacity in obese individuals. Cardiopulmonary exercise test is currently the international gold standard for evaluating patients’ exercise ability and cardiopulmonary function, which provides an objective assessment of maximum aerobic capacity, hemodynamic responses, electrocardiogram (ECG), VE/VCO_2,_ VO_2_/HR, and breathing reserve (BR) (Santamaría-Ulloa, Chinnock, and Montero-López, 2022). Although numerous studies have explored the association between obesity and various health risks, there remains a limited in-depth analysis of the exercise endurance and cardiopulmonary function characteristics of obese patients (Mechanick et al., 2020; Lavie, Milani, and Ventura, 2009; Jackson et al., 2015). Hao et al. (2022) conducted a cross-sectional analysis on obese patients aged 40–60 with varying degrees of obesity, revealing differences in blood pressure and ventilation efficiency between the obese and normal groups during CPX. However, this study did not explore indicators related to aerobic exercise capacity, such as VO2_peak_ and VO2_AT_, and the age distribution of the study population was also different. The aim of our study was to evaluate the exercise ability and cardiopulmonary function of obese people through cardiopulmonary exercise test, and compare the differences in exercise ability and cardiopulmonary function of patients with different degrees of obesity. We hypothesize that as the degree of obesity increases, the participants’ maximum exercise capacity and cardiorespiratory function also decline.

## 2 Materials and methods

### 2.1 Participants and study design

Data were collected from September 2019 to January 2024 at the Department of Rehabilitation Medicine of the Affiliated Wuxi People’s Hospital of Nanjing Medical University. This study was conducted in accordance with the Declaration of Helsinki. Protocols involving human participants were reviewed and approved by the Institutional Ethics Committee of the Affiliated Wuxi People’s Hospital of Nanjing Medical University (KS2019020). All participants were evaluated with cardiopulmonary exercise tests, which was performed using the same equipment by the same experienced doctors, who are senior attending physicians with qualifications in cardiopulmonary exercise test operation and interpretation for more than 8 years. The participants in the overweight and obesity groups were required to undergo cardiopulmonary exercise testing as part of their weight intervention plan, while participants in the control group were individuals with normal BMI who came for a health checkup. Inclusion criteria were as follows: (1) ages 18–60 years; (2) body mass index (BMI) ≥18.5 kg/m^2^; (3) available cardiopulmonary exercise test (CPET) data; (4) signed the informed consent form. Exclusion criteria were as follows: (1) cardiovascular, respiratory or neuroskeletal diseases that affect the implementation of cardiopulmonary exercise tests; (2) having rheumatic and immune diseases, hematological disorders, or malignant tumors; (3) patients with secondary obesity, including diseases such as hypothyroidism, Cushing’s syndrome, or long-term use of drugs that cause obesity; (4) participants were taking any medications that might affect cardiopulmonary responses to exercise, such as β receptor blockers. Before undergoing a cardiopulmonary exercise test, signing an informed consent form was a crucial step. This ensured that the patient fully understood the purpose, procedure, potential risks, and benefits of the test, and agreed to participate voluntarily. On the informed consent form, it was essential to indicate the exclusion criteria related to cardiovascular, respiratory, rheumatology, and immunology diseases, allowing the patient to check whether they had these conditions. This ensured the safety and effectiveness of the test. Finally, a total of 231 participants were included into the cross-sectional study. According to the obesity classification criteria of the World Health Organization (WHO) (Do et al., 2018), taking into account the particularities of the Chinese population, they were categorized into five groups based on their BMI: the control group (18.5 ≤ BMI < 24 kg/m^2^) with 28 cases, the overweight group (24.0 ≤ BMI < 28 kg/m^2^) with 48 cases, the mild obesity group (28 ≤ BMI < 35 kg/m^2^) with 75 cases, the moderate obesity group (35.0 ≤ BMI < 40 kg/m^2^) with 47 cases, and the severe obesity group (BMI ≥ 40 kg/m^2^) with 33 cases,” based on the Chinese standards (Zeng et al., 2021) and the Expert Consensus on the Prevention and Treatment of Obesity in Chinese Adults released by the Obesity Group of the Chinese Medical Association Endocrinology Branch in 2011.

In this study, to ensure statistical power and the reliability of the results, we utilized the PASS 15.0 software for sample size estimation. Based on the results of the preliminary trial, we selected the mean VO2_peak_/kg values for five groups of participants with different BMI levels to estimate the sample size. We set the significance level (α) at 0.05 and the statistical power (1-β) at 0.90. The results indicated that under these conditions, at least 23 participants per group were required to achieve the desired statistical power. We then compiled the baseline data (such as gender composition, age, height, and exercise habits) of a total of 231 participants whose complete data were collected from September 2019 to January 2024. We found that the number of participants included in each of the five groups was greater than 23 (n = 28/48/75/47/33), and there were no statistically significant differences in the baseline data of these 231 participants across the five groups (P < 0.05). Therefore, a total of 231 participants were ultimately included.

### 2.2 Retrospective

This study presented a retrospective analysis of data derived from subjects who fulfilled the specified inclusion and exclusion criteria and underwent cardiopulmonary exercise testing at the Rehabilitation Department of Wuxi People’s Hospital affiliated with Nanjing Medical University, spanning from September 2019 to January 2024.

### 2.3 Cardiopulmonary exercise test

The test was performed by the CARDIOVIT CS-200 Excellence ErgoSpiro System (Schiller, Switzerland). The participants underwent a symptom-limited cardiopulmonary exercise test. A trained professional rehabilitation physician performed a physical examination, history, and cardiopulmonary exercise test measurements. All the participants signed informed consent forms. Prior to the cardiopulmonary exercise test, we assessed the safety of participants’ exercise through the PAR-Q scale (Physical Activity Readiness Questionnare) (Smith et al., 2024) and evaluated their exercise habits by verbal inquiry. Participants who exercised for at least 3 days a week for 3 months or more were considered to have an exercise habit. Firstly, preheated the machine for 15 min after startup, and then performed environmental temperature calibration, humidity calibration, capacity calibration, gas calibration, etc. in sequence. Following electrode placement for a 12-lead ECG, a blood pressure cuff was applied to the upper arm for the duration of the test and participants were fitted with a respiratory facemask for expiratory gas analysis. Prior to beginning the exercise test, resting ECG, blood pressure, and pulmonary function measures were obtained. The static pulmonary function measures included FEV1, FEV1%, FVC,FVC%, FEV1/FVC, PEF. We measured the maximum voluntary ventilation of each participant in a quiet state. Then, adjusted the seat to the proper height. The participants remained at rest for 3 min until the data of the machine was stable. The exercise test lasted for approximately 20 min for each participant with 3-minutes of warm-up, 8–12 for the graded exercise test, and 3-min of cool-down. The revolutions per minute of the power bicycle was maintained at (60 ± 5) r/min. All participants used the bicycle ergometer protocol, with a starting wattage of 0. The average incremental wattage per minute was calculated by dividing the patient’s predicted maximum wattage by 10. Blood oxygen saturation, heart rate, electrocardiogram and pulmonary ventilation indexes were dynamically monitored throughout the exercise, and blood pressure was measured every 2 min. The anaerobic threshold was determined by the V-slope method (Beaver, Wasserman, and Whipp, 1986; Nishijima et al., 2017). In our study, the anaerobic threshold was automatically calculated by the Cardiovit system. In the absence of an oxygen consumption plateau, we determined the maximal effort level of the CPET by the RPE reaching between 17 and 19. Additionally, we ensured that each participant’s RER_max_ was greater than 1.15 to confirm the maximal effort level of the exercise test.

### 2.4 Observation index

The observation indexes were as follows:VO_2_ at anaerobic threshold (VO_2AT_), percentage of predicted VO_2AT_ (VO_2AT_% Pred), peak oxygen consumption (VO_2peak_), percentage of predicted VO_2peak_ (VO_2peak_% Pred), peak kilogram oxygen consumption (VO_2peak_/kg), maximum exercise power (WR_max_), breathing reserve (BR), maximum heart rate (HR_max_), percentage of predicted HR_max_ (HR_max_% Pred), maximum O_2_ pulse (VO_2_/HR_max_), percentage of predicted maximum O_2_ pulse (VO_2_/HR_max_%Pred), maximum relative O_2_ pulse (VO_2_/HR_max_/kg), forced vital capacity (FVC), ratio of forced expiratory volume to vital capacity in 1 s (FEV1/FVC), percentage of predicted forced vital capacity (FVC% Pred), percentage of predicted forced expiratory volume ratio of 1 s (FEV1% Pred), peak expiratory flow rate (PEF),maximum exercise ventilation (VE_max_), maximum voluntary ventilation (MVV),Heart rate response (HRr)= (HRmax-HRrest)/(VO_2peak_-VO_2rest_). The respiratory reserve was automatically calculated by the system based on the actual ventilation volume during the exercise and resting MVV.

### 2.5 Statistical analysis

SPSS 26.0 statistical software was used for data analysis. Continuous variables with measurement data consistent with normal distribution were represented by mean ± standard deviation; one-way ANOVA was used for comparison of the mean values of each indicator among groups; LSD method was used for comparison of homogeneity of variance; Tambane’s T2 method was used for variance heterogeneity. Continuous variables that do not adhere to a normal distribution were typically represented by the median and the interquartile range (IQR, P25,P75). To compare these variables across different groups, the Kruskal–Wallis test was employed. Spearman correlation analysis was used to analyze the correlation between BMI and each indicator; *P* < 0.05 was considered statistically significant.

## 3 Result

### 3.1 Comparison of demographic data for groups

The demographic data of the five groups were compared as follows. and there was no statistical significance in gender composition, age, height, and exercise habit of the five groups of patients (*P* > 0.05). There were statistically significant differences in body mass index (BMI) and body mass among the five groups of participants (*P* < 0.05) (Table 1).

**TABLE 1:** Comparison of general information for groups.

Groups	Cases	Gender		Age (years old)	height (cm)	weight (kg)	BMI (kg/m²)	Exercise habit	
		Male	Female					Yes	No
control	28	13	15	31.50 (28.00.36.00)	165.46 ± 6.403	60.25 ± 7.271	22.45 (20.30, 23.30)	14	14
overweight	48	29	19	33.50 (30.00,41.75)	168.23 ± 6.359	74.03 ± 6.598	26.20 (25.43, 27.18)	25	23
mild obesity	75	52	23	32.00 (29.00,44.00)	169.63 ± 7.080	90.08 ± 9.393	31.20 (29.70, 32.80)	27	48
moderate obesity	47	31	16	32.00 (26.00,38.00)	171.38 ± 10.479	109.95 ± 15.150	37.30 (35.80, 38.70)	17	30
severe obesity	33	16	17	29.00 (25.00.38.50)	168.73 ± 8.857	124.15 ± 16.790	43.40 (41.30, 45.90)	13	20
χ2 or F or H		χ2 = 7.356		H = 6.892	F = 2.677	F = 182.203	H = 216.985	4.590	
*p*-value		0.118		0.142	0.033	<0.001	<0.001	0.332	

### 3.2 Comparison of exercise cardiopulmonary test indexes for groups

Compared to the control group and the overweight group, the VO_2AT_ levels were higher in the mild obesity group, moderate obesity group and severe obesity group. Statistically significant differences were observed in the overall distribution of VO_2AT_ across the five groups. Post-hoc analyses revealed statistically significant differences between the overweight group and both the mild obesity group, the moderate obesity group, and the severe obesity group. Additionally, a significant difference was noted between the control group and the moderate obesity group, as well as between the control group and the severe obesity group.

Significant differences were identified in the overall distribution of VO_2AT_/kg among the five groups, as well as in the overall distribution of VO_2peak_. Among these, significant differences were observed between the control group and the moderate obesity group, the overweight group and the moderate obesity group, and the overweight group and the severe obesity group.

Significant differences were noted in the overall distribution of VO_2AT_/kg among the five groups. Specifically, statistically significant differences were found between the control group and the overweight group, the mild obesity group, the moderate obesity group, and the severe obesity group.

**FIGURE 1:**
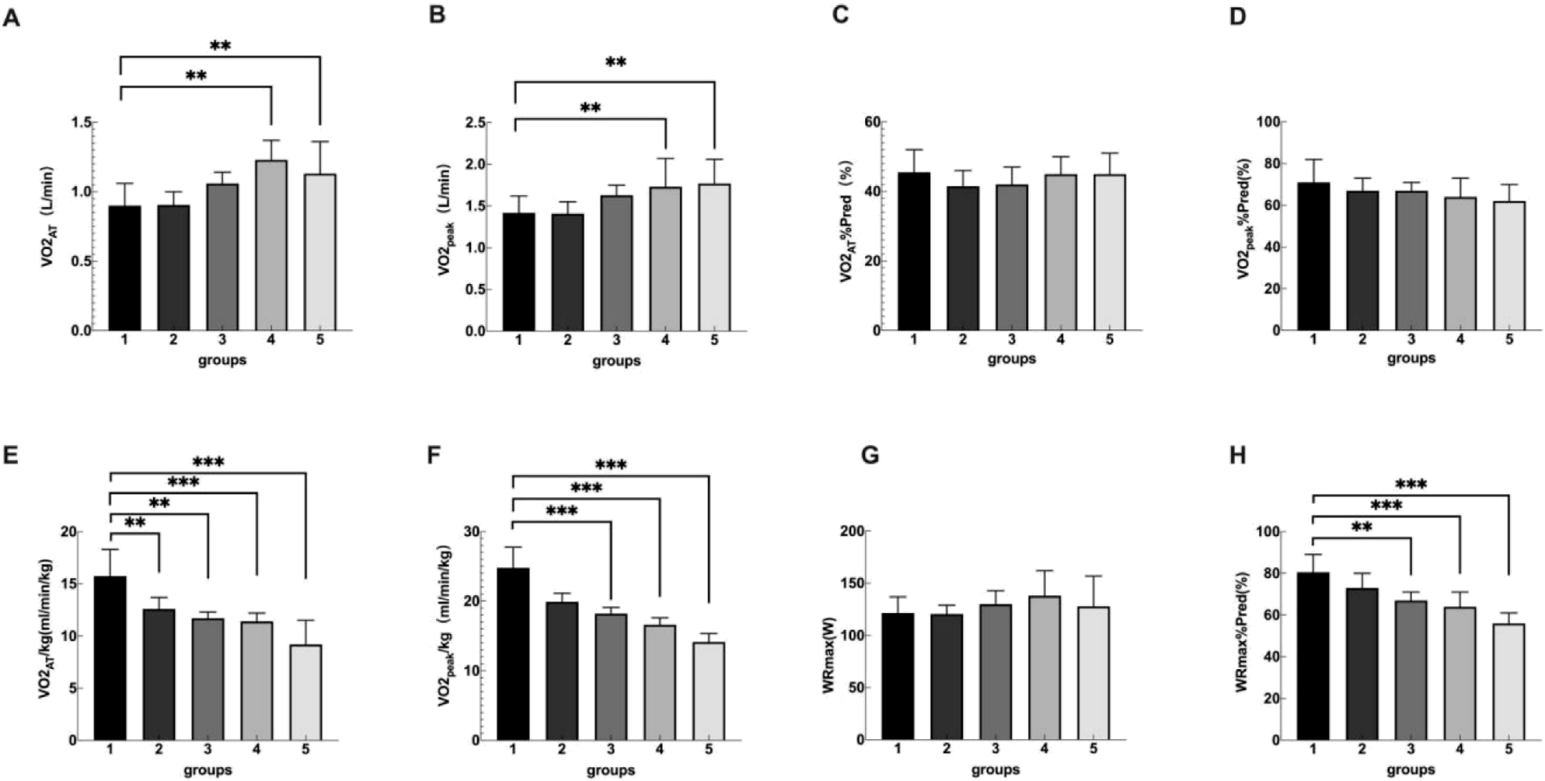
Group 1, 2, 3, 4, 5 represents the control group, overweight group, mild obesity group, moderate obesity group, and sever obesity group, respectively. The comparison of cardiopulmonary exercise test indexes for five groups on VO2_AT_ **(A)**, VO2_max_ **(B)**, VO2_AT_/Pred **(C)**, VO2_max_/Pred **(D)**, VO2_AT_/kg **(E)**, VO2_max_/kg **(F)**, WR_max_ **(G)**, WR_max_/Pred **(H)**. **p < 0.05, ***p < 0.001 vs. Control group. ns means no statistically significant difference compared to the control group.

Significant differences were identified in the overall distribution of VO2_peak_/kg among the five groups. The differences between the control group and the mild obesity group, the moderate obesity group, and the severe obesity group were statistically significant. No significant differences were detected in WR_max_ among the five groups. However, the overall distribution of WR_max_%Pred showed significant differences among the five groups, with notable differences between the control group and the obesity group (Table 2; Figure 1).

**TABLE 2:** Comparison of cardiopulmonary exercise test indexes for groups.

Groups	VO_2AT_ (L/min)	VO_2AT_/Pred (%)	VO_2peak_ (L/min)	VO_2peak_/Pred (%)	VO_2AT_/kg (mL/min/kg)	VO_2peak_/kg (ml/min/kg)	WR_max_ (W)	WR_max_/Pred (%)
Control	0.90 (0.79, 1.16)	45.50 (38.50, 57.00)	1.42 (1.29, 1.62)	71.00 (58.50, 87.50)	15.75 (12.60, 19.10)	24.25 (20.13, 30.83)	121.50 (105.25, 153.75)	80.50 (70.00, 91.00)
Overweight	0.91 (0.75, 1.08)	41.50 (32.25, 48.00)	1.41 (1.33, 1.71)	67.00 (55.00, 76.00)	12.60** (9.85, 14.76)	19.85 (16.88, 23.80)	120. (105.25, 142.50)	73.00 (64.00, 83.75)
Mild obesity	1.06 (0.96, 1.19)	42.00 (35.00, 51.00)	1.63 (1.42, 1.85)	67.00 (55.00, 76.00)	11.70** (10.50, 13.60)	18.50*** (16.00, 20.80)	130.00 (110.00, 156.00)	67.00** (60.00, 77.00)
Moderate obesity	1.23** (1.02, 1.49)	45.0 (35.00, 54.00)	1.73** (1.49, 2.22)	64.00 (55.00, 76.00)	11.40*** (10.20, 13.40)	16.50*** (14.80, 18.90)	138.00 (115.00, 174.00)	64.00*** (55.00, 75.00)
Severe obesity	1.13** (0.97, 1.59)	45.00 (36.50, 53.00)	1.77** (1.39, 2.21)	62.00 (56.50, 73.00)	9.20*** (7.80, 12.15)	13.60*** (11.50, 17.20)	128.00 (0.70, 0.91)	56.00*** (51.50, 66.00)
H	37.370	4.538	23.018	3.008	34.747	66.606	7.079	45.136
*p*-value	<0.001	0.338	<0.001	0.556	<0.001	<0.001	0.132	<0.001

Note: **p < 0.05,***p < 0.001 vs. Control group.

**FIGURE 2:**
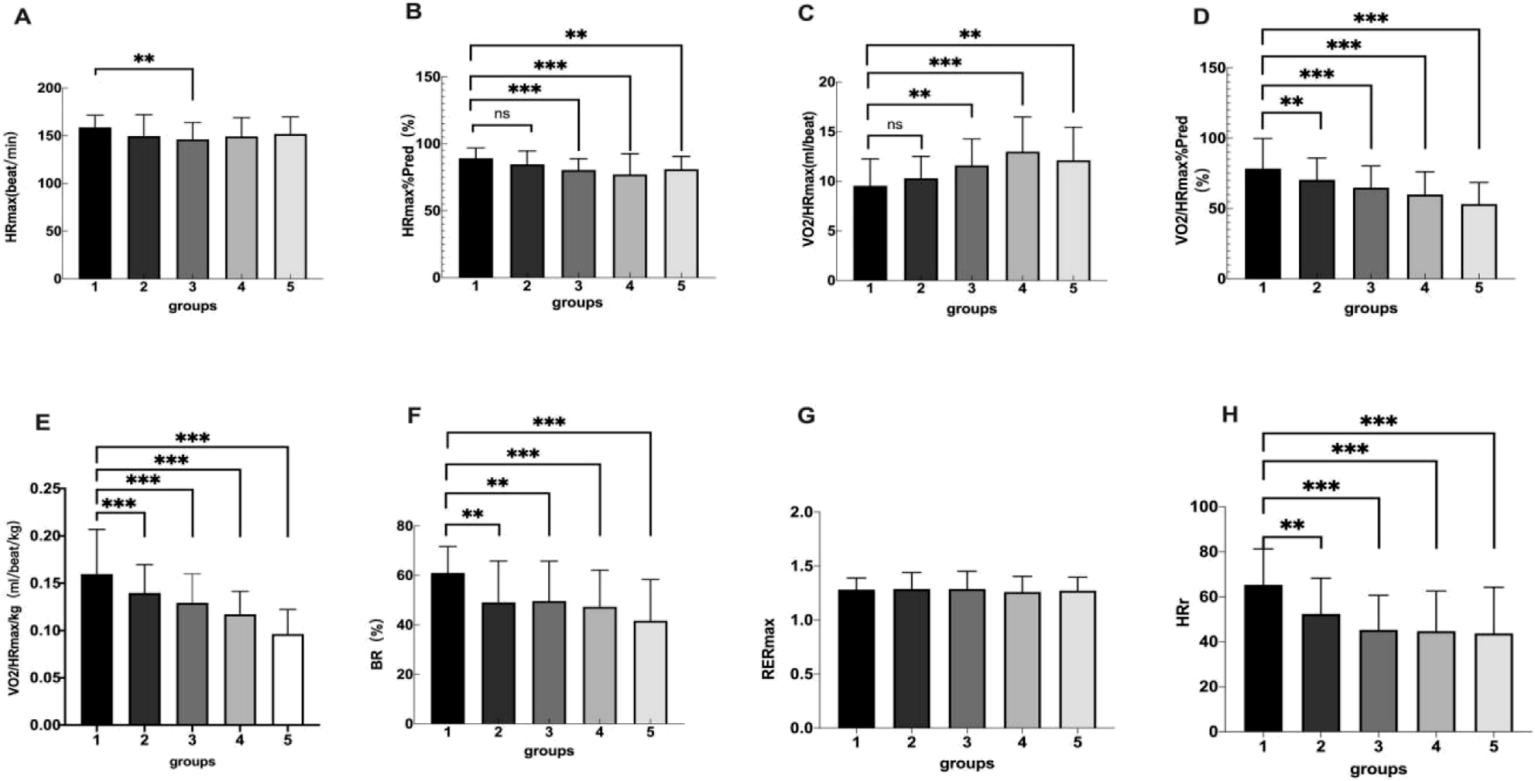
Group 1, 2, 3, 4, 5 represents the control group, overweight group, mild obesity group, moderate obesity group, and sever obesity group, respectively. The comparison of cardiopulmonary exercise test indexes for five groups on HR_max_ **(A)**, HR_max_/Pred **(B)**, VO2/HR_max_ **(C)**, VO2/HR_max_%Pred **(D)**, BR **(E)**, RER_max_ **(F)**, HRr **(G).** **p < 0.05, ***p < 0.001 vs. Control group.

There were statistical significant difference among the five groups in HR_max_, HR_max_%Pred, VO_2_/HR_max_, VO_2_/HR_max_%Pred, VO_2_/HR_max_/kg, BR and HRr. There were no significant differences among the five groups in RER_max_ (Table 3; Figure 2).

**TABLE 3:** Comparison of cardiopulmonary exercise test indexes for groups.

Groups	HR_max_ (1/min)	HR_max_%Pred (%)	VO_2_/HR_max_ (ml/beat)	VO_2_/HR_max_ %Pred (%)	VO_2_/HR_max_/kg (ml/beat/kg)	RER_max_	BR (%)	HRr
Control	158.71 ± 12.78	89.17 ± 7.71	9.54 ± 2.71	78.43 ± 21.36	0.16 ± 0.05	1.28 ± 0.11	61.00 ± 10.74	65.21 ± 15.95
Overweight	149.75 ± 22.20	84.65 ± 10.01	10.292 ± 2.21	70.50 ± 15.46**	0.14 ± 0.03***	1.28 ± 0.15	49.15 ± 16.67**	52.39 ± 15.80**
Mild obesity	146.09 ± 17.90 **	80.47 ± 8.41***	11.60 ± 2.67**	64.79 ± 15.46***	0.13 ± 0.03***	1.28 ± 0.16	49.63 ± 16.12**	45.29 ± 15.27***
Moderate obesity	149.34 ± 19.62	77.06 ± 15.51***	12.98 ± 3.50***	60.02 ± 16.16***	0.12 ± 0.02***	1.25 ± 0.14	47.34 ± 14.82***	44.72 ± 17.84***
Severe obesity	151.97 ± 17.76	81.00 ± 9.48**	12.12 ± 3.34**	53.27 ± 15.33***	0.10 ± 0.03***	1.27 ± 0.12	41.70 ± 16.63***	43.69 ± 20.55***
F	2.443	6.920	8.803	11.354	18.688	0.336	6.147	9.467
*p*-value	0.048	<0.001	<0.001	<0.001	<0.001	0.854	<0.001	<0.001

Note: **p < 0.05,***p < 0.001 vs. Control group.

### 3.3 Comparison of static pulmonary function

**FIGURE 3:**
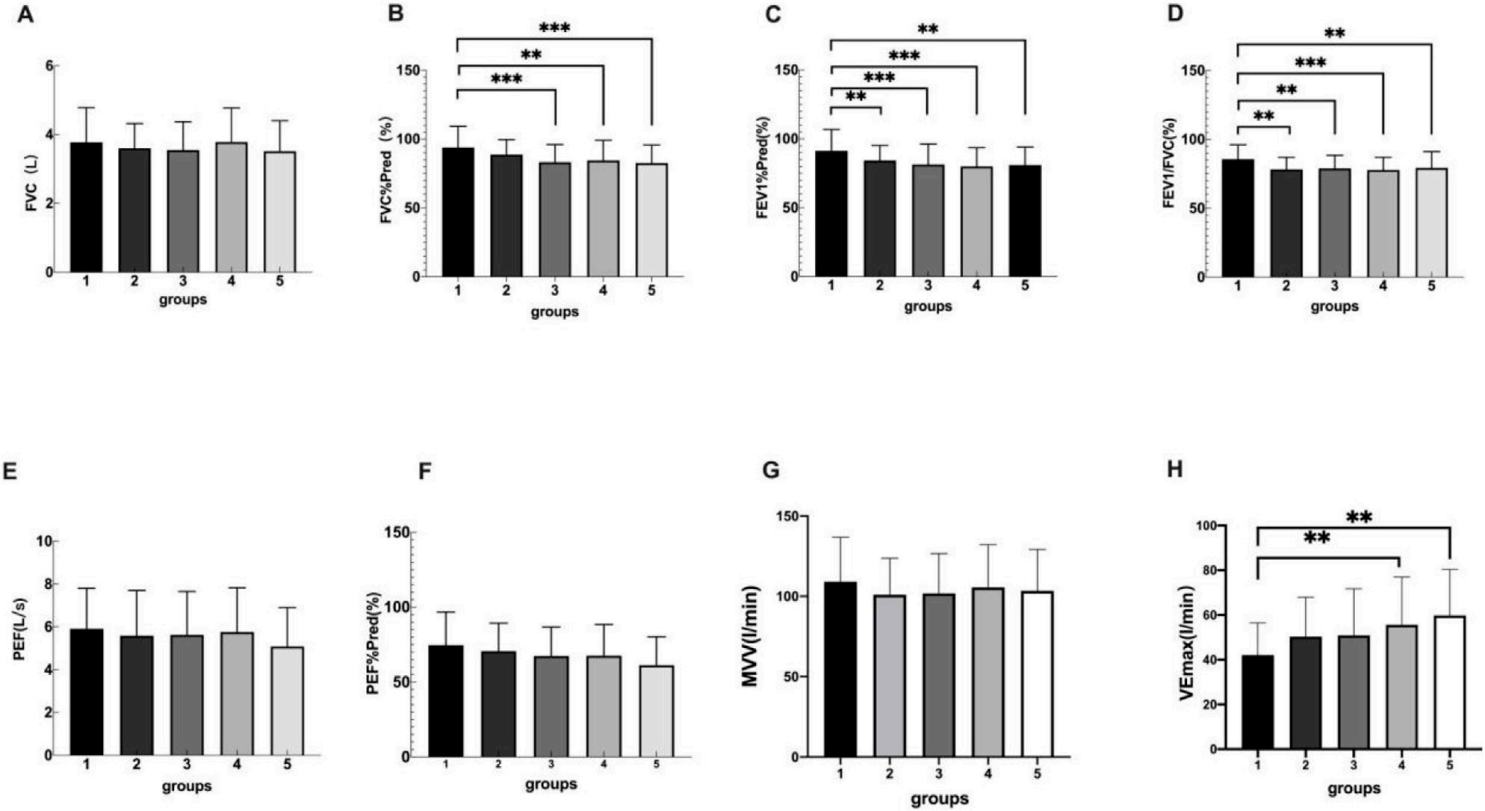
Group 1, 2, 3, 4, 5 represents the control group, overweight group, mild obesity group, moderate obesity group, and sever obesity group, respectively. The comparison of cardiopulmonary exercise test indexes for five groups on FVC **(A)**, FVC%Pred **(B)**, FEV1%Pred **(C)**, FEV1/FVC **(D)**, PEF **(E)**, PEF%Pred **(F).** **p < 0.05, ***p < 0.001 vs. Control group.

There were significant differences among the five groups in FVC%Pred, FEV1%Pred and FEV1/FVC. FEV1/FVC in overweight and obesity groups were lower than those in control group (*P* < 0.05), but there was no significant difference between obesity groups (*P* > 0.05). There was a statistically significant difference in PEF%Pred in severe obesity vs. control group, overweight vs. severe obesity, and there was no statistically significant difference between obesity groups (*P* > 0.05). There was no differences among the five groups in MVV(*P* > 0.05), and there were significant differences among the five groups in VE_max_ (*P* = 0.005) (Table 4; Figure 3). The multiple comparisons among the various indicators of cardiopulmonary exercise test are shown in Table 5.

**TABLE 4:** Comparison of static pulmonary function.

Groups	FVC (L)	FVC%Pred (%)	FEV1%Pred (%)	FEV1/FVC (%)	PEF (L/s)	PEF%Pred (%)	MVV (L/min)	VE_max_ (L/min)
Control	3.77 ± 1.00	93.89 ± 15.43	91.25 ± 15.50	85.64 ± 10.37	5.91 ± 1.89	74.71 ± 22.13	109.08 ± 27.66	37.63 (32.80, 48.81)
Overweight	3.60 ± 0.72	88.81 ± 10.81	84.33 ± 10.94**	78.31 ± 8.67**	5.59 ± 2.11	70.69 ± 18.67	100.97 ± 22.75	47.41 (37.54, 64.95)
Mild obesity	3.55 ± 0.82	83.23 ± 12.72***	81.41 ± 14.76***	78.93 ± 9.58**	5.63 ± 2.03	67.48 ± 19.16	101.95 ± 24.61	47.92 (36.82, 60.26)
Moderate obesity	3.78 ± 0.99	84.49 ± 14.74**	79.94 ± 13.60***	77.87 ± 9.13***	5.76 ± 2.07	67.60 ± 20.85	105.64 ± 26.51	50.72 (40.60, 70.89)**
Severe obesity	3.51 ± 0.89	82.61 ± 13.01***	81.06 ± 12.91**	79.33 ± 11.81**	5.09 ± 1.82	61.36 ± 18.96	103.56 ± 25.64	59.62 (44.87, 71.39)**
F or H	0.858	4.577	3.681	3.343	0.787	1.973	0.620	14.983
*p*-value	0.49	0.001	0.006	0.011	0.535	0.99	0.65	0.005

Note: **p < 0.05,***p < 0.001 vs. Control group.

**TABLE 5:** Intergroup multiple comparisons of cardiopulmonary exercise test data indicators

Intergroup comparison		VO_2AT_		VO_2peak_		VO_2AT_/kg		VO_2peak_/kg	
group (I)	group (J)	mean difference (I-J)	P	mean difference (I-J)	P	mean difference (I-J)	P	mean difference (I-J)	P
control	overweight	8.760	1.000	−1.266	1.000	50.613	0.014	40.802	0.102
	mild obesity	−29.403	0.469	−33.148	0.251	59.405	0.001	63.513	<0.001
	moderate obesity	−63.144	0.001	−55.321	0.005	67.305	<0.001	91.138	<0.001
	severe obesity	−53.735	0.017	−45.032	0.087	98.511	<0.001	125.000	<0.001
overweight	mild obesity	−38.164	0.020	−31.881	0.098	8.972	1.000	22.711	0.660
	moderate obesity	−71.904	<0.001	−54.054	0.001	16.692	1.000	50.336	0.002
	severe obesity	−62.495	<0.001	−43.766	0.038	47.898	0.015	84.198	<0.001
mild obesity	moderate obesity	−33.740	0.066	−22.173	0.745	7.901	1.000	27.625	0.263
	severe obesity	−24.332	0.813	−11.885	1.000	39.106	0.051	61.487	<0.001
moderate obesity	severe obesity	9.409	1.000	10.288	1.000	31.205	0.398	33.862	0.257
Intergroup comparison		WR_max_/Pred		HR_max_		HR_max_%Pred		VO_2_/HR_max_	
group (I)	group (J)	mean difference (I-J)	P	mean difference (I-J)	P	mean difference (I-J)	P	mean difference (I-J)	P
control	overweight	28.055	0.774	8.964	0.251	4.533	0.074	−0.749	0.918
	mild obesity	55.499	0.002	12.621	0.002	8.712	<0.001	−2.056	0.012
	moderate obesity	70.647	<0.001	9.374	0.136	12.115	<0.001	−3.436	<0.001
	severe obesity	101.565	<0.001	6.745	0.614	8.179	0.003	−2.575	0.015
overweight	mild obesity	27.444	0.263	3.657	0.984	4.179	0.034	−1.307	0.038
	moderate obesity	42.592	0.019	0.41	1.000	7.582	0.001	−2.687	<0.001
	severe obesity	73.510	<0.001	−2.22	1.000	3.646	0.13	−1.827	0.079
mild obesity	moderate obesity	15.148	1.000	−3.247	0.988	3.403	3.403	−1.380	−1.380
	severe obesity	46.067	0.010	−5.876	0.719	−0.533	−0.533	−0.520	−0.520
moderate obesity	severe obesity	30.918	0.416	−2.629	1.000	−3.936	0.104	0.861	0.957
Intergroup comparison		VO_2_/HR_max_ %Pred		VO_2_/HR_max_/kg		BR		HRr	
group (I)	group (J)	mean difference (I-J)	P	mean difference (I-J)	P	mean difference (I-J)	P	mean difference (I-J)	P
control	overweight	7.929	0.043	0.021	0.355	11.854	0.002	12.815	0.002
	mild obesity	13.642	<0.001	0.031	0.025	11.373	0.001	19.916	<0.001
	moderate obesity	18.407	<0.001	0.043	0.001	13.660	<0.001	20.491	<0.001
	severe obesity	25.156	<0.001	0.063	<0.001	19.303	<0.001	21.520	<0.001
overweight	mild obesity	5.713	0.061	0.010	0.521	−0.481	0.867	7.101	0.023
	moderate obesity	10.479	0.002	0.022	0.002	1.805	0.572	7.676	0.027
	severe obesity	17.2279	<0.001	0.042	<0.001	7.449	0.035	8.705	0.023
mild obesity	moderate obesity	4.765	0.120	0.012	0.169	2.286	0.430	0.574	0.855
	severe obesity	11.51394*	0.001	0.032	<0.001	7.930	0.015	1.603	0.649
moderate obesity	severe obesity	6.749	0.071	0.020	0.007	5.643	0.111	1.029	0.788
Intergroup comparison		FVC%Pred		FEV1%Pred		FEV1/FVC		VE_max_	
group (I)	group (J)	mean difference (I-J)	P	mean difference (I-J)	P	mean difference (I-J)	P	mean difference (I-J)	P
control	overweight	5.080	0.107	6.917	0.034	7.330	0.002	−32.448	0.412
	mild obesity	10.666*	<0.001	9.837	0.001	6.710*	0.002	−32.538	0.279
	moderate obesity	9.403	0.003	11.314*	0.001	7.771	0.001	−45.444	0.044
	severe obesity	11.287	0.001	10.189	0.004	6.310	0.013	−63.504	0.002
overweight	mild obesity	5.586	0.023	2.920	0.248	−0.621	0.731	−0.090	1.000
	moderate obesity	4.323	0.112	4.397	0.118	0.440	0.826	−12.996	1.000
	severe obesity	6.206	0.039	3.273	0.290	−1.021	0.644	−31.056	0.399
mild obesity	moderate obesity	−1.263	0.607	1.477	0.561	1.061	0.560	−12.906	1.000
	severe obesity	0.621	0.822	0.353	0.902	−0.400	0.845	−30.965	0.265
moderate obesity	severe obesity	1.883	0.530	−1.124	0.717	−1.461	0.510	−18.060	1.000

### 3.4 Correlation analysis between BMI and cardiopulmonary exercise test indexes

In terms of cardiopulmonary exercise ability, BMI was positively correlated with VO_2AT_,VO_2peak,_ VE_max_ and VO_2_/HR_max_, and negatively correlated with VO_2AT_/kg, VO_2peak_/kg,WR_max_%Pred, HR_max_%Pred, VO_2_/HR_max_%Pred, VO_2_/HR_max_/kg,BR and HRr. In terms of static pulmonary function, BMI was negatively correlated with FVC%Pred, FEV1%Pred, and PEF%Pred (Table 6).

**TABLE 6:** Correlation analysis between BMI and cardiopulmonary exercise test indexes.

Items	BMI	
	Correlation coefficient (r)	*p* values
VO_2AT_	0.384**	<0.001
VO_2peak_	0.281**	<0.001
VO_2AT_%Pred	0.060	0.365
VO_2peak_%Pred	−0.123	0.062
VO_2AT_/kg	−0.362**	<0.001
VO_2peak_/kg	−0.569**	<0.001
WR_max_	0.117	0.076
WR_max_%Pred	−0.450**	<0.001
HR_max_	−0.068	0.305
HR_max_%Pred	−0.260**	<0.001
VO_2_/HR_max_	0.308**	<0.001
VO_2_/HR_max_%Pred	−0.460**	<0.001
VO_2_/HR_max_/kg(ml/beat/kg)	−0.521**	<0.001
RER_max_	−0.068	0.305
BR	−0.265**	<0.001
FVC	−0.023	0.733
FVC%Pred	−0.242**	<0.001
FEV1%Pred	−0.214**	0.001
FEV1/FVC	−0.091	0.170
PEF	−0.082	0.214
PEF%Pred	−0.173**	0.009
HRr	−0.0335**	<0.001
MVV (L/min)	0.000	0.996
VE_max_ (L/min)	0.224**	0.001

Note: **p < 0.01,*p < 0.05: the correlation was significant.

VO_2AT_, VO_2_ at anaerobic threshold; VO_2AT_% Pred, percentage of predicted VO_2AT_; VO_2peak_, peak oxygen consumption; VO_2peak_% Pred :percentage of predicted VO_2peak_; VO_2peak_/kg, peak kilogram oxygen consumption; WR_max_ :maximum exercise power; BR, breathing reserve, BR, (MVV-peak VE)/MVV(%); HR_max_, maximum heart rate; HR_max_% Pred, percentage of predicted HR_max_; VO_2_/HR_max_, maximum O_2_ pulse; VO_2_/HR_max_%Pred :percentage of predicted maximum O_2_ pulse; FVC, forced vital capacity; FEV1/FVC, ratio of forced expiratory volume to vital capacity in 1 second; FVC%, Pred :percentage of predicted forced vital capacity; FEV1%, Pred :percentage of predicted forced expiratory volume ratio of 1 second; PEF, peak expiratory flow rate; PEF%Pred, percentage of predicted peak expiratory flow rate; HRr, Heart rate response, HRr, (HRmax-HRrest)/(VO_2peak_-VO_2rest_).

## 4 Discussion

Cardiopulmonary exercise capacity refers to the ability of the human heart, lungs, and vascular system to efficiently transport oxygen and nutrients to muscle tissues during exercise, while effectively removing metabolic waste. This capability is one of the strongest predictors of the risk for future adverse events in apparently healthy individuals (Guazzi et al., 2018). The study results revealed that VO2_AT_ rose in the obesity group compared to individuals with normal or overweight BMI, and this increase correlated with the severity of obesity. VO_2AT_ serves as a crucial indicator of aerobic capacity, reflecting the muscle mitochondria’s ability to utilize oxygen (Masuda et al., 2022). Given the more pronounced weight differences between groups compared to height differences, the rise in BMI among obese patients is primarily attributed to an increase in body mass, which necessitates greater oxygen consumption under the same exercise load. This study results indicated that as BMI increases, VO_2AT_/kg gradually decreases. There is a significant difference between obesity groups and control group. As obesity worsens, the increase in VO_2_ among obese patients does not keep pace with the increase in body weight, leading to a decline in VO_2AT_/kg among obese patients. This aligns with the correlation analysis results of this study, which show that BMI is positively correlated with VO_2AT_ and negatively correlated with VO_2AT_/kg. This phenomenon may be attributed to the following mechanisms: ①Decreased muscle mass: Obese individuals often exhibit a reduction in muscle mass, particularly in skeletal muscle (Khalid et al., 2022). Given that muscle is the primary organ for oxygen consumption, a decrease in muscle mass results in a diminished overall oxygen consumption capacity. ②Elevated adipose tissue: As obesity progresses, the body accumulates more adipose tissue. Although adipose tissue is not the primary site of oxygen consumption, its increase contributes to the overall body weight, thereby lowering the oxygen consumption per kilogram of body weight (VO_2AT_/kg). ③Deteriorated cardiovascular function: Obese patients frequently suffer from compromised cardiovascular function, including weakened heart pumping capacity and heightened vascular resistance. These factors impair the efficiency of oxygen transport and utilization, leading to a reduction in VO2_AT_/kg. ④Suboptimal metabolic efficiency: Obese individuals may experience reduced metabolic efficiency, such as mitochondrial dysfunction and decreased oxidative phosphorylation efficiency (de Mello et al., 2018). These factors hinder the body’s ability to efficiently utilize oxygen, resulting in a decline in VO2_AT_/kg. The difference in VO_2AT_%Pred among the five groups was not statistically significant, and the median values were all within the normal range (percentage of VO_2AT_ to predicted value >40%) (Guazzi et al., 2012). This is considered because the patients included in this study were young to middle-aged adults, and the anaerobic threshold appeared at a moderate to low exercise intensity, thus allowing the VO2_AT_%Pred of all five groups to remain within the normal range.

In terms of maximum exercise capacity,VO_2peak_ is a index that quantifies maximum exercise capacity and is widely used as a standard to assess the effectiveness of rehabilitation interventions (Toulouse et al., 2021). The results of this study showed that the VO_2peak_ of the three groups of obese patients was higher than that of the non-obese patients, but the VO_2peak_%Pred and VO_2peak_/kg gradually decreased with the increase of obesity degree. The results of Marta Gruchała--Niedoszytko et al. were similar Gruchała-Niedoszytko et al. (2019). Battista et al. (2023) have shown that BMI is an independent determinant of low VO_2peak_/kg. Ross et al. (2016) research suggested that compared to VO_2peak_, VO_2peak_/kg is more reflective of daily living ability and represents the strongest long-term prognostic indicator of disability and mortality. VO_2peak_ is predominantly influenced by arterial blood oxygen content, maximum cardiac output, muscle oxygen capacity, and the exercise muscle distribution index, representing the upper limit of the body’s oxygen supply capacity (Mikkelsen et al., 2020). In this study, no significant difference was observed in WR_max_ across the five groups; however, the overall distribution of WR_max_%Pred exhibited significant variations among the groups. The WR_max_%Pred of the three obese patient groups was lower than that of the control group. To match the exercise load of non-obese individuals, obese patients must exert greater effort. Chinese obese patients predominantly exhibit abdominal obesity (Zhao et al., 2018), with increased fat weight, normal or reduced muscle mass, heightened oxygen demand, reduced muscle oxygen utilization rate, relatively inadequate oxygen supply, intensified anaerobic glycolysis, lactic acid accumulation, and diminished exercise capacity (Ren et al., 2021). The correlation analysis conducted in this study further revealed that BMI exhibited a positive correlation with VO_2AT_ and VO_2peak_, while demonstrating a negative correlation with VO_2peak_%Pred, VO_2AT_/kg, VO_2peak_/kg, and WR_max_%Pred. Consequently, the author posits that a patient’s exercise capacity cannot be solely determined by a high VO_2peak_.Instead, a comprehensive evaluation that includes VO_2peak_%Pred, VO_2peak_/kg, and cardiopulmonary examination results should be considered.

In this study, no significant differences were observed in RER_max_ across the five patient groups, with RER_max_ exceeding 1.2, indicating that the exertion levels of the five groups of patients were satisfactory. The VO_2_/HR_max_ for the three obesity groups was higher than that of the control group, while HR_max_%Pred and VO_2_/HR_max_%Pred were lower compared to the control group. Correlation analysis further revealed that BMI was negatively correlated with HR_max_%Pred and VO_2_/HR_max_%Pred, aligning with the findings of Gonze et al. (2021). VO_2_/HR_max_ represents the product of the heart’s stroke volume and the arterial-mixed venous blood oxygen content difference [C (a-v)O2], which is positively correlated with cardiac output and serves as an indicator of cardiac function (Jürgensen et al., 2015). At the commencement of the incremental test, as exercise intensity increases, stroke volume escalates to meet the body’s demand for blood and oxygen, leading to a corresponding rise in oxygen pulse (Kilding, Sequeira, and Wood, 2018). In this study, the oxygen pulse of patients with mild to moderate obesity was higher than that of the control group; however, the maximum oxygen pulse of the severely obesity group was lower compared to the mild and moderate obesity group. The maximum relative oxygen pulse (Christou et al., 2021) of obese and overwight groups were lower compared to the control group,indicating that the metabolic level of the heart is lower during exercise. The primary consideration for this is the impact of cardiac fat deposition on cardiac contraction and pumping function following the progression of obesity, resulting in a decrease in stroke volume (Tutor et al., 2023). This confirms the rapid increase in cardiac output in obese patients and the adverse biomechanics that lead to cardiovascular inefficiency. Heart rate response (HRr) refers to the changes in heart rate that occur in response to various physiological and psychological stimuli, which is related to exercise tolerance (Ishihara et al., 2019). In this study, the HRr of overweight and obese participants were lower than that of the control group, and the differences were statistically significant, indicating a reduced exercise tolerance in obese individuals. Obese patients exhibited significantly lower BR compared to the control group. It is considered that to meet the physiological demands during exercise, obese patients increase their respiratory rate and minute ventilation, which makes their respiratory muscles more prone to fatigue (Chlif et al., 2017), thereby reducing breathing reserve. Research has shown that breathing reserve is negatively correlated with body weight (Opina et al., 2019), which aligns with the negative correlation between BMI and BR observed in this study. The differences in BMI among the patients in this study were primarily due to variations in weight, as there were no significant differences in height.

It is important to note that the oxygen pulse varies with different levels of obesity and should not be solely relied upon to judge good cardiac function; instead, it should be interpreted in conjunction with VO_2_/HR_max_%Pred and other indicators to comprehensively assess the cardiac function through cardiopulmonary exercise testing, determining whether the patient is in a compensatory state. Similarly, a high VO_2peak_ should not be the sole criterion to judge good exercise capacity; it is advisable to integrate VO_2peak_%Pred, VO_2peak_/kg, and other factors for a comprehensive evaluation of the patient’s exercise capacity.

Taking into account that VO_2peak_ values differed, but WR_max_ did not differ between individuals of various BMI categories, it suggests that individuals of various BMI are capable of achieving similar levels of work (power output) but are utilizing different amounts of oxygen to do so. This discrepancy highlights potential differences in exercise economy, which can be attributed to several factors related to body composition, metabolic efficiency, and biomechanics. People who are overweight or obese have higher levels of adipose tissue. Adipose tissue can interfere with the efficiency of oxygen delivery to muscles and lead to higher resting metabolic rates, both of which can reduce exercise economy. Normal weight individuals may have more lean muscle mass, which can enhance metabolic efficiency. Muscle tissue is more metabolically active than adipose tissue, meaning it can produce more work per unit of oxygen consumed. People with high BMI have lower VO2/HR_max_/kg. This may limit the amount of oxygen-rich blood delivered to the working muscles, requiring higher unit oxygen consumption. In addition, overweight and obese individuals have lower respiratory mechanical efficiency, resulting in higher ventilation rates relative to VO_2_. This may lead to a decrease in the efficiency of oxygen use during exercise. Higher body mass can increase the load on joints, potentially reducing mechanical efficiency. This can lead to higher oxygen consumption to achieve the same power output. Overweight and obese individuals may have more difficulty dissipating heat during exercise due to increased adipose tissue insulation. This can lead to higher core temperatures and increased cardiovascular strain, further reducing exercise economy.

In terms of static pulmonary function, FVC and FVC%Pred gradually decrease with the increase of BMI. Considering the mechanical load caused by the accumulation of excess adipose tissue in chest wall and abdomen of obese patients, the compliance of respiratory system, functional residual capacity and expiratory reserve capacity are poor, and the activity of diaphragm decreases (Jones and Nzekwu, 2006). The breathing reserve of obese patients was lower than that of normal patients, and was negatively correlated with the degree of obesity.

Compared to previous studies, this research focused on a younger population of obese adults and conducted a more systematic analysis of the aerobic exercise capacity, maximal exercise capacity, cardiac function, and pulmonary function characteristics of individuals with varying degrees of obesity. It was found that as the degree of obesity increases, patients experienced a decrease in FVC and BR, as well as a reduction in HRr. BMI shows a positive correlation with VO2_AT_ and VO2_peak_, and a negative correlation with VO2_peak_%Pred, VO2_AT_/kg, VO2_peak_/kg, and WR_max_%Pred.

Through this study, we learned that as BMI increases, participants’ cardiorespiratory function is negatively affected. Therefore, when we encounter obese patients in clinical practice, we attach great importance to their obesity issues. We educate patients on the importance of addressing obesity and encourage them to take proactive weight management measures to prevent further harm caused by obesity. At the same time, through cardiopulmonary exercise testing, we can identify the exercise risks of obese patients, ensure their exercise safety, and develop accurate exercise prescriptions that are more suitable for patients.

## 5 Conclusion

In summary, although the exercise capacity of obese patients remains within the normal range, as the degree of obesity increases and exercise intensity escalates, their cardiopulmonary function and maximum exercise capacity decline.

## 6 Study limitation

The limitation of this study cannot be ignored. First,the data collection was conducted at a single center, with a small sample size, which may introduce selection bias. In future studies, we could opt for multi-center sampling.Second, not all participants reached the the VO_2max_,which is a better indicator of an individual's maximum exercise capacity compared to VO_2peak_.Third, Blood sampling and lactate measurement during exercise testing can provide a better understanding of exercise metabolic changes.Fourth,as this article is a retrospective study, not every participant has undergone human body composition analysis. Therefore, we were unable to analyze VO_2_ in lean body mass. But in future research, we will analyze VO_2_ in lean body mass more accurately instead of VO2/kg. However, due to the limitations of our experimental conditions, we were unable to do this.

## Data Availability

The original contributions presented in the study are included in the article/supplementary material, further inquiries can be directed to the corresponding authors.
