# Supplementary material for: Characteristics of cardiopulmonary exercise capacity in adults with different degrees of obesity
Source: Front Physiol. 2025 Jan 20;15:1466153. doi: 10.3389/fphys.2024.1466153 (PMC11788284; doi:10.3389/fphys.2024.1466153)
Supplement: Supplementary file 1 [file DataSheet1.pdf]

# *Supplementary Material*

## *Figures and tables(This article has 5 tables and 3 figures)*

Table 1. Comparison of general information for groups

| Groups | Cases | Gender | | Age(years old) | height （cm） | weight(kg) | Exercise habit | |
|---|---|---|---|---|---|---|---|---|
| | | Male | Female | | | | yes | no |
| control | 28 | 13 | 15 | 31.50（28.00,36.00） | 165.46 ± 6.403 | 60.25 ± 7.271 | 14 | 14 |
| overweight | 48 | 29 | 19 | 33.50（30.00,41.75） | 168.23 ± 6.359 | 74.03 ± 6.598 | 25 | 23 |
| mild obesity | 75 | 52 | 23 | 32.00（29.00,44.00） | 169.63 ± 7.080 | 90.08 ± 9.393 | 27 | 48 |
| moderate obesity | 47 | 31 | 16 | 32.00（26.00,38.00） | 171.38 ± 10.479 | 109.95 ± 15.150 | 17 | 30 |
| severe obesity | 33 | 16 | 17 | 29.00（25.00.38.50） | 168.73 ± 8.857 | 124.15 ± 16.790 | 13 | 20 |
| $\chi 2$ or F or H | | $\chi 2$=7.356 | | H=6.892 | F=2.677 | F=182.203 | 4.590 | |
| p-value | | 0.118 | | 0.142 | 0.033 | ＜0.001 | 0.332 | |

Table 2. Comparison of cardiopulmonary exercise test indexes for groups

| groups | $VO2_{AT}$ (L/min) | $VO2_{AT}$/Pred (%) | VO2max (L/min) | VO2max/Pred (%) | $VO2_{AT}$/kg (ml/min/kg) | VO2max/kg (ml/min/.kg) | $WR_{max}$ (W) | $WR_{max}$/Pred (%) |
|---|---|---|---|---|---|---|---|---|
| control | 0.90 (0.79,1.16) | 45.50 (38.50,57.00) | 1.42 (1.29,1.62) | 71.00 (58.50,87.50) | 15.75 (12.60,19.10) | 24.25 (20.13,30.83) | 121.50 (105.25,153.75) | 80.50 (70.00,91.00) |
| overweight | 0.91 (0.75,1.08) | 41.50 (32.25,48.00) | 1.41 (1.33,1.71) | 67.00 (55.00,76.00) | 12.60** (9.85,14.76) | 19.85 (16.88,23.80) | 120. （105.25,142.50） | 73.00 (64.00,83.75) |
| mild obesity | 1.06 (0.96,1.19) | 42.00 (35.00,51.00) | 1.63 (1.42,1.85) | 67.00 (55.00,76.00) | 11.70** (10.50,13.60) | 18.50*** (16.00,20.80) | 130.00 (110.00,156.00) | 67.00** (60.00,77.00) |
| moderate obesity | 1.23** (1.02,1.49) | 45.0 (35.00,54.00) | 1.73** (1.49,2.22) | 64.00 (55.00,76.00) | 11.40*** (10.20,13.40) | 16.50*** (14.80,18.90) | 138.00 (115.00,174.00) | 64.00*** (55.00,75.00) |
| severe obesity | 1.13** (0.97,1.59) | 45.00 (36.50，53.00) | 1.77** (1.39,2.21) | 62.00 (56.50,73.00) | 9.20*** (7.80,12.15) | 13.60*** (11.50,17.20) | 128.00 (0.70,0.91) | 56.00*** (51.50,66.00) |
| H | 37.370 | 4.538 | 23.018 | 4.197 | 34.747 | 66.606 | 7.079 | 45.136 |
| P-value | < 0.001 | 0.338 | < 0.001 | 0.380 | < 0.001 | < 0.001 | 0.132 | < 0.001 |

Note: **p < 0.05,***p < 0.001 vs. Control group

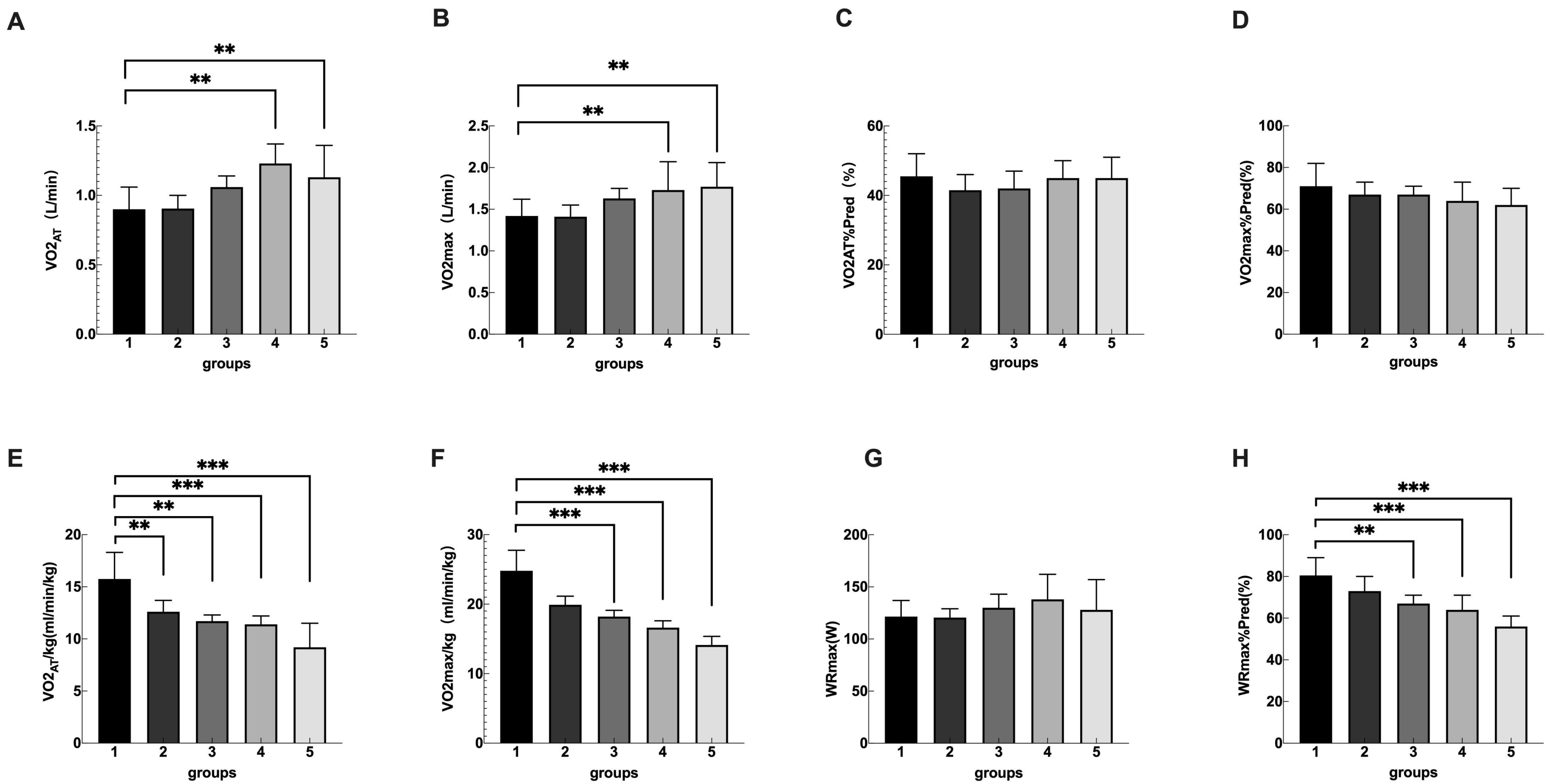


**Figure 1**

Group 1,2,3,4,5 represents the control group, overweight group, mild obesity group, moderate obesity group, and severe obesity group, respectively. The comparison of cardiopulmonary exercise test indexes for five groups on $VO2_{AT}$(A), VO2max(B), $VO2_{AT}$/Pred(C), VO2max/Pred(D), $VO2_{AT}$/kg(E), VO2max/kg(F), WRmax(G), WRmax/Pred(H).

**$p < 0.05$,***$p < 0.001$ vs. Control group.ns means no statistically significant difference compared to the control group.

Table 3. Comparison of cardiopulmonary exercise test indexes for groups

| Groups | $HR_{max}$ (1/min) | $HR_{max}$%Pred (%) | $VO_2/HR_{max}$ (ml/beat) | $VO_2/HR_{max}$ %Pred （%） | $RER_{max}$ | BR (%) | HRr |
|---|---|---|---|---|---|---|---|
| control | 158.71±12.78 | 89.17±7.71 | 9.54±2.71 | 78.43±21.36 | 1.28±0.11 | 61.00±10.74 | 65.21±15.95 |
| overweight | 149.75±22.20 | 84.65±10.01 | 10.292±2.21 | 70.50±15.46** | 1.28±0.15 | 49.15±16.67** | 52.39±15.80** |
| mild obesity | 146.09±17.90 ** | 80.47±8.41*** | 11.60±2.67** | 64.79±15.46*** | 1.28±0.16 | 49.63±16.12** | 45.29±15.27*** |
| moderate obesity | 149.34±19.62 | 77.06±15.51*** | 12.98±3.50*** | 60.02±16.16*** | 1.25±0.14 | 47.34±14.82*** | 44.72±17.84*** |
| severe obesity | 151.97±17.76 | 81.00±9.48** | 12.12±3.34** | 53.27±15.33*** | 1.27±0.12 | 41.70±16.63*** | 43.69±20.55*** |
| F | 2.443 | 6.920 | 8.803 | 11.354 | 0.336 | 6.147 | 9.467 |
| P-value | 0.048 | < 0.001 | < 0.001 | < 0.001 | 0.854 | < 0.001 | < 0.001 |

Note: **p < 0.05,***p < 0.001 vs. Control group

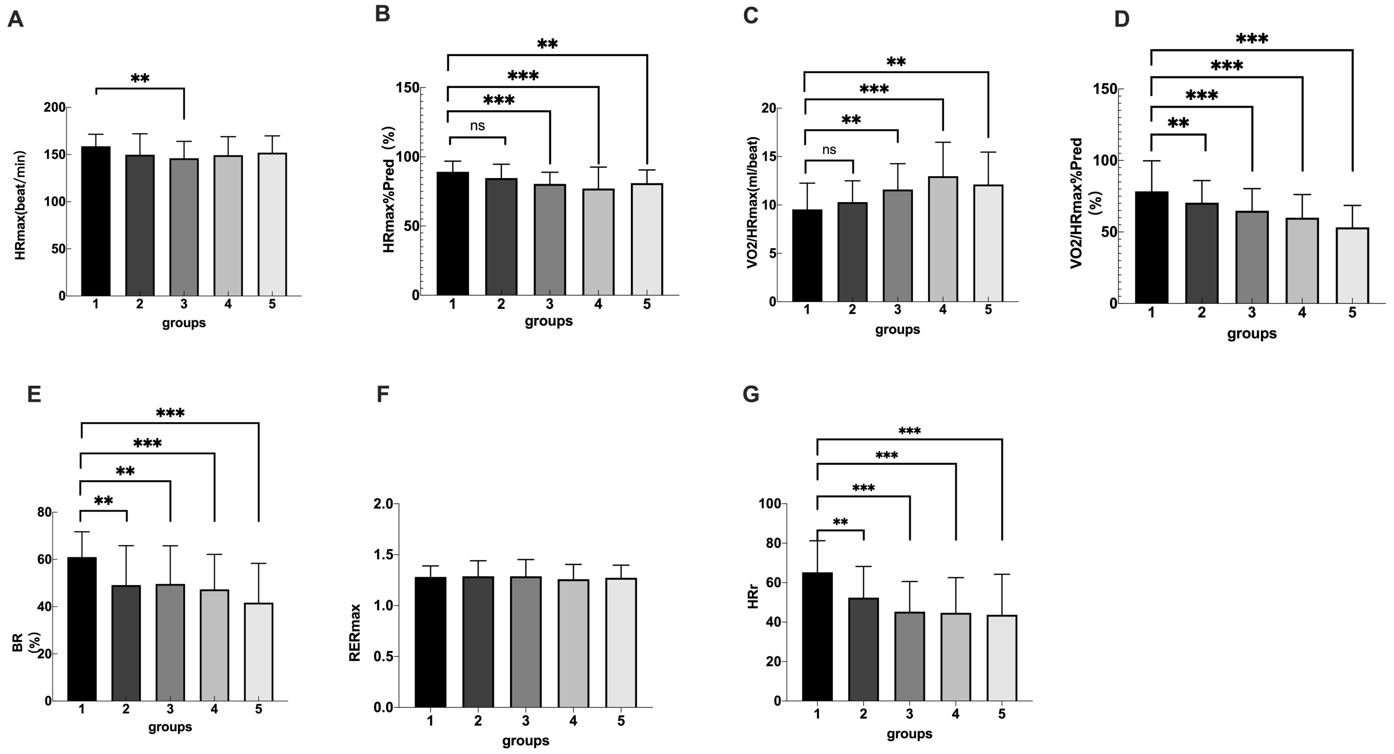


**Figure 2**

Group 1,2,3,4,5 represents the control group, overweight group, mild obesity group, moderate obesity group, and severe obesity group, respectively. The comparison of cardiopulmonary exercise test indexes for five groups on HRmax(A), HRmax%Pred(B), VO2/HRmax (C),VO2/HRmax %Pred (D), BR(E), RERmax(F), HRr(G).

**$p < 0.05$,***$p < 0.001$ vs. Control group.

Table 4. Comparison of static pulmonary function

| Groups | FVC (L) | FVC%Pred（%） | FEV1%Pred（%） | FEV1/FVC（%） | PEF(L/s) | PEF%Pred（%） |
|---|---|---|---|---|---|---|
| control | 3.77±1.00 | 93.89±15.43 | 91.25±15.50 | 85.64±10.37 | 5.91±1.89 | 74.71±22.13 |
| overweight | 3.60±0.72 | 88.81±10.81 | 84.33±10.94** | 78.31±8.67** | 5.59±2.11 | 70.69±18.67 |
| mild obesity | 3.55±0.82 | 83.23±12.72*** | 81.41±14.76*** | 78.93±9.58** | 5.63±2.03 | 67.48±19.16 |
| moderate obesity | 3.78±0.99 | 84.49±14.74** | 79.94±13.60*** | 77.87±9.13*** | 5.76±2.07 | 67.60±20.85 |
| severe obesity | 3.51±0.89 | 82.61±13.01*** | 81.06±12.91** | 79.33±11.81** | 5.09±1.82 | 61.36±18.96 |
| F | 0.858 | 4.577 | 3.681 | 3.344 | 0.787 | 1.979 |
| P-value | 0.49 | 0.001 | 0.006 | 0.011 | 0.535 | 0.99 |

Note: **p < 0.05,***p < 0.001 vs. Control group

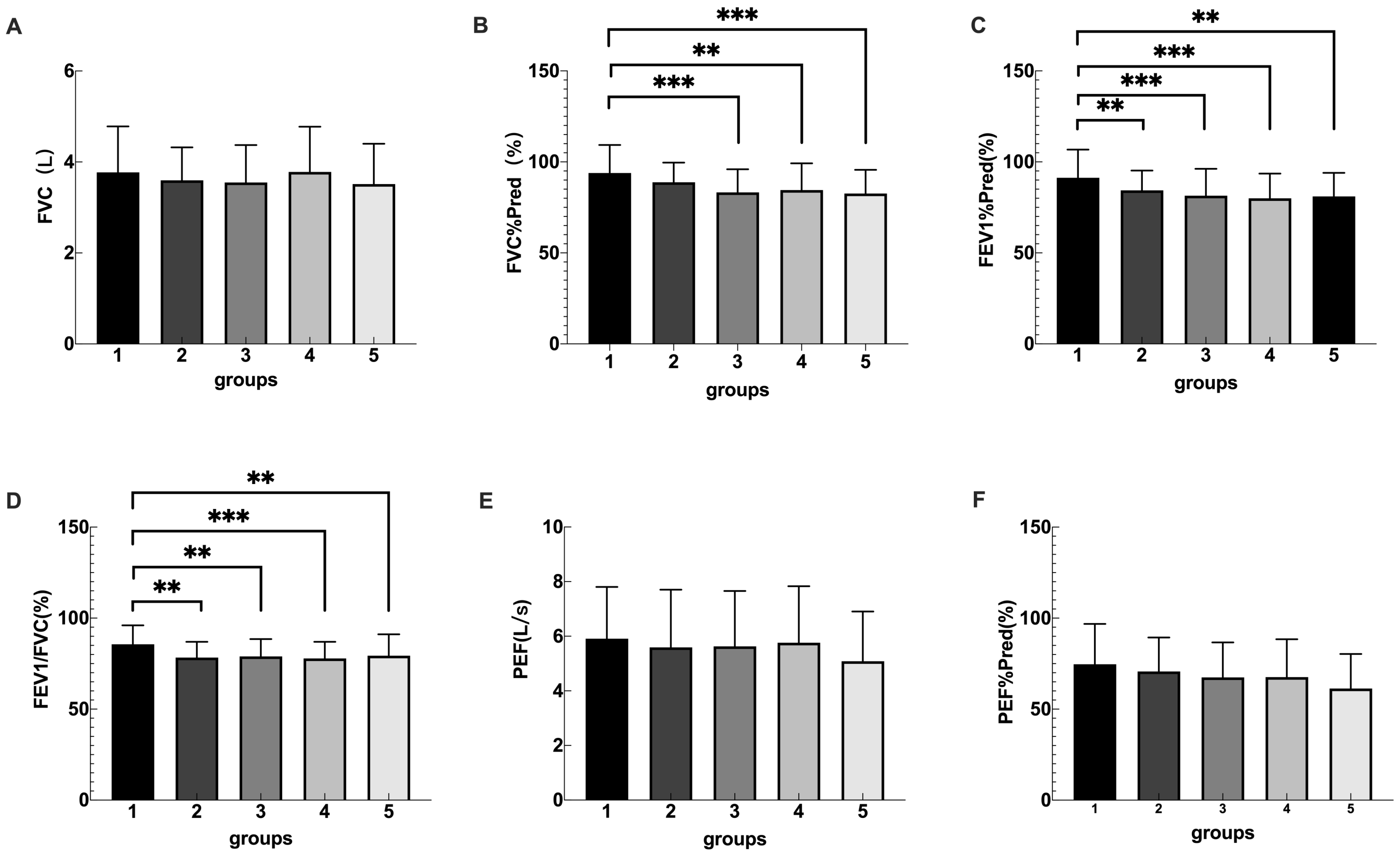


**Figure 3**

Group 1,2,3,4,5 represents the control group, overweight group, mild obesity group, moderate obesity group, and severe obesity group, respectively.

The comparison of cardiopulmonary exercise test indexes for five groups on FVC (A), FVC%Pred(B), FEV1%Pred(C), FEV1/FVC(D), PEF(E), PEF%Pred(F).

**$p < 0.05$,***$p < 0.001$ vs. Control group.

Table 5 .Correlation analysis between BMI and cardiopulmonary exercise test indexes

| items | BMI | |
|---|---|---|
| | Correlation coefficient (r) | p values |
| $VO_{2AT}$ | 0.384** | < 0.001 |
| $VO_{2max}$ | 0.281** | < 0.001 |
| $VO_{2AT}$%Pred | 0.060 | 0.365 |
| $VO_{2max}$%Pred | -0.123 | 0.062 |
| $VO_{2AT}$/kg | -0.362** | < 0.001 |
| $VO_{2max}$/kg | -0569** | < 0.001 |
| $WR_{max}$ | 0.117 | 0.076 |
| $WR_{max}$%Pred | -0.450** | < 0.001 |
| $HR_{max}$ | -0.068 | 0.305 |
| $HR_{max}$%Pred | -0.260** | < 0.001 |
| $VO_2$/HRmax | 0.308** | < 0.001 |
| $VO_2$/HRmax%Pred | -0.460** | < 0.001 |
| $RER_{max}$ | -0.068 | 0.305 |
| BR | -0.265** | < 0.001 |
| FVC | -0.023 | 0.733 |
| FVC%Pred | -0.242** | < 0.001 |
| FEV1%Pred | -0.214** | 0.001 |
| FEV1/FVC | -0.091 | 0.170 |
| PEF | -0.082 | 0.214 |
| PEF%Pred | -0.173** | 0.009 |
| HRr | -.0335** | <0.001 |

Note:**p < 0.01,*p < 0.05 : the correlation was significant .
